# Oral health policymaking challenges in Iran: a qualitative approach

**DOI:** 10.1186/s12903-020-01148-w

**Published:** 2020-06-01

**Authors:** Mohammadtaghi Mohammadpour, Peivand Bastani, David Brennan, Arash Ghanbarzadegan, Jamshid Bahmaei

**Affiliations:** 1 Student Research Committee, Shiraz University of Medical Sciences, Shiraz, Iran; 2 Health Human Recourses Research Center, School of Health Management and Medical Informatics, Shiraz University of Medical Sciences, Shiraz, Iran; 3 Australian Research Centre for Population Oral Health (ARCPOH), Adelaide Dental School, University of Adelaide, Adelaide, South Australia Australia

**Keywords:** Oral health, Developing country, Policymaking, Strategy

## Abstract

**Background:**

As the strategies proposed for oral health improvement in developed countries are not adapted for developing ones, this study aimed to identify the challenges of oral health policy implementation in Iran as a low-income developing country.

**Methods:**

This qualitative study was conducted in 2019 in Iran as a middle-eastern developing country. The study population consisted of experts who had experience in oral health and were willing to participate in the study. Snowball sampling was used to select 12 participants for semi-structured interviews and saturation was achieved after 16 interviews. Guba and Lincoln criteria including credibility, transferability, confirmability and dependability were used to determine reliability and transparency, and finally a five-step framework analysis method was used to analyze the data.

**Results:**

The analysis of the interviews resulted in identification of 7 main themes that were categorized into 5 problems of policy implementation as proposed by the Matus framework. The main themes of executive and preventive challenges to implement oral health policies were categorized as organizational problems, the main themes of educational and resource challenges were situated as material problems, and the main themes of insurance, policy making and trusteeship challenges were considered as legal, policymaking and perspective.

**Conclusion:**

The implementation of oral health policies has faced some challenges. It seems that the national coverage of oral health and integration of these services in prevention and serious attention to the private sector can be considered as the most important strategies for achieving improved oral health in Iran.

## Introduction

In spite of the World Health Organization (WHO) report for everyone’s health by 2000 as well as significant progress in many fields of public health, there has been little success in the prevention and improvement of oral health and the burden of diseases is increasing; this is referred to as the silent epidemic [1]. A study on the burden of diseases in 2017 showed that oral diseases affect more than half of the world’s population and untreated caries in children was estimated as 560 million cases worldwide [2].

Iran is a low income developing country. According to the latest National Oral Health Survey in 2012, the number of Decayed, Missed and Filled Teeth (DMFT) in children aged 5–6 years was 5.16 in the whole country. This index has been reported to be 4.94 and 5.78 in urban and rural areas, respectively [2]. Furthermore, another national report showed that Iranian school children’s DMFT was twice the world’s standard [3].

Considering that achieving oral health in different communities poses a great economic burden; the poor condition of oral health in Iran becomes more significant. In this regard, a national survey of household expenditure in Iran showed that 15.5% of the total household health expenditure was related to oral health [4]. These statistics are important regarding the lack of basic benefit packages and insurance coverage to pay the costs of oral diseases in Iran [5]. In addition, evidence suggests that poor oral health may lead to consequences, including difficulties in swallowing, sleeping, socializing and social well-being in children [6]. Previous studies have also shown that improving oral health can lead to improved communication, increased quality of life, as well as increased self-esteem and social confidence [6, 7]. There are many factors contributing to poor oral health, some of which may include inadequate self-care, lack of access to oral health services, socio-economic factors, and personal obstacles and problems [7–9] as well as policy challenges.

Policy making is a process that refers to the manner by which policies are created, arranged, implemented and evaluated and for this purpose this process contains four important steps of problem identification and issue recognition, policy formulation, policy implementation and policy evaluation [10] that we focused on according to the Matus (1996) framework. Matus has pointed to five aspects of material, organizational, political, logical, and the perspective of policies that affect the implementation of the policy and leads to their products or impacts [11].

There are different policies in oral health according to the facilities of the countries and based on their policies, various policy implementation approaches have been applied. In this regard, WHO as an international organization that has the responsibility for global health, has declared some policy priorities for improving oral health as follows: effective use of fluoride; healthy nutrition; tobacco use control; and improvements in the oral health of children and adolescents’ through school, elderly oral health, the oral health system, Human immunodeficiency virus infection and acquired immune deficiency syndrome (HIV/AIDS) and oral health, oral health information systems, and oral health research [12]. One of the strategies taken to implement these policies is to integrate oral health care with primary health care, as a leading strategy for accessing oral health care services for children and adolescents including fluoride therapy, dentist visits, and caries prevention [13].

Another strategy for oral health policy implementation includes the organization of the workforce who are responsible for improving community oral health. In this regard, Yamalik et al. [14] has compared the policymaking and workforce planning of developed and developing countries. These results showed that the median number of dentists, the numbers of dental practices, dental hygienists, technicians and graduates per year was greater in developed countries [14]. These indicators can lead to a different oral health status among these countries and it also emphasizes that the strategies proposed for developed countries may not work as well in developing ones and globally [15]. So it is necessary to find local strategies in order to achieve acceptable coverage of oral health, particularly in low and middle income developing countries.

According to the above-mentioned points, it seems that different communities have different concerns of policy implementation and need different practical policies and strategies for achieving effective prevention in the field of oral health as well as reducing inequality and improving coverage of high-risk age groups. At the same time, reviewing the history of the interventions in Iran, shows that in spite of implementing the policies of integrating oral health services in the primary health care (PHC) plan for about two decades and achievements in improving some oral health indicators in rural areas, this policymaking experienced a serious failure because of neglecting to train sufficient technicians and lack of a tendency among dentists to deliver services in such areas [16].

In sum, it seems that it is important to review the challenges of local policies in order to shed light for policy makers and healthcare managers to find applied solutions for implementing policies or correcting them. So, this study was conducted to identify the challenges and barriers of oral health policy implementation in Iran as a low-income developing country in the Middle East.

## Methods

The present study was a qualitative one conducted in 2019 using content analysis. We used semi-structured interviews to achieve a diversity of views that included national and local policymakers, oral health professors, regional oral health managers and assistants involved in this field (Table 1). The reason for choosing these participants is that Iran has a centralized policymaking approach through which oral health policies are developed by Ministry of Health and Medical Education. The policies are sent to oral health offices affiliated to medical universities all over the country to be localized and implemented.

**Table 1:** The characteristics of participants in the study

Relationship of participants to oral health	Number
Head of oral health department	1
Experts of regional oral health department	3
Health assistant of the University	1
Head of social dentistry department	1
Head of health policy making department	1
Social dentists professors	4
Head of dentistry school	1

The purpose of performing these interviews was to explain the challenges of policy implementation in the field of oral health and the reasons for neglecting the complete implementation of oral health policies in spite of the upstream documents of the Islamic Republic of Iran. The interviewees were selected by snowball sampling method. First, the Oral Health Officer of Shiraz University of Medical Sciences as the metropolitan area of the southern part of the country and his deputies were interviewed and then asked to identify those who were experts in the field among the other regions of the country (Table 1).

The study participants were well-informed and experienced in the field of oral health and policymaking or management and executive tasks; they spoke well and were willing to share their information. At this stage of the work, the interviews were performed with the participants after arrangements were made with them in person and preferably at their workplace. At the beginning of the interview, explanations were provided verbally about the study and its purpose, and they were assured about the confidentiality of their information. A written informed consent form was also obtained from all the participants, and they were assured that they were free to withdraw from the study at any stage of the research if they did not wish to continue. The interviews lasted for at least 50 min and all the interviews were performed by one of the researchers (JB). All the interviews were recorded by a voice recorder with the consent of the participants and transcribed word by word shortly after the interview. All the audios and transcriptions were stored on an external hard drive and all of them were labeled numerically in order to maintain the confidentiality of the verbal and written data. The interviews continued until saturation and after performing 16 interviews with 12 interviewees, saturation was reached. For determining this saturation level, we had 2 separate interviews with 4 of the participants that had provided the most information and cooperation with the research team. In other words, in the qualitative approach the level of saturation determines the data collection stop point and the participants that can be included in a session according to the level of information they have and their tendency to share the related information [17].

In order to provide a semi-structured interview, a topic guide consisting of 10 questions was used based on literature review and two pilot interviews with the managers and policy experts in the Shiraz University of Medical Sciences. After that, the initial draft of the topic guide was prepared. The meaningfulness and face validity of the questions were verified through performing three initial interviews with the interviewees to finalize the topic guide. The questions are presented in Table 2.

**Table 2:** The main questions of the topic guide

What do you think about the oral health policy implementation in the country? Are they successful or not?	
In your opinion what are the most problems and barriers in oral health policy implementation in the country?	
Can you give some examples of the problems in the way of implementing oral health policies?	
Can you differentiate among the problems in the scopes of prevention, treatment and education?	
Which of these three do you think need more consideration or even reform?	
What do you think about the resources? What kind of resources you think are essential for implementing oral health policies?	
What do you think about the role of insurance packages?	
How about the regional executive problems, can you exemplify some problems in this area.	
How about the policies? In your opinion are all the problems associated with the inappropriate policy implementations or policymaking as well? (the same as defining the agenda, etc.)	
How about the structure, organization or other infrastructures? How can you illustrate their role?	
If there is any other concept you want to point that was not mentioned in the previous questions please talk about them.	

After data transcription, a five-stage framework analysis method was used to analyze the data. In order to identify the data at the first stage, the audio files of the interview meetings were listened to several times by the researcher and the texts were read several times. At the second stage, in order to identify a thematic framework, the repeated ideas in the familiarization process were transformed into groups of similar ideas or codes. These codes were achieved via an explicit and implicit extraction of the transcriptions according to the Matus (1996) framework [11]. At the third stage, indexing, units, or parts of the data associated with a particular code were identified. At the fourth stage, after indexing, the data were summarized as a code table based on the thematic framework, and finally at the fifth stage, the data were finally combined, mapped and interpreted to define the concepts and show the relationship between them to identify the nature of the phenomenon and provide explanations and suggestions [18]. The data were coded and categorized manually instead of using software because of the Persian text of the interviews and to enhance creativity. In order to insure the validity, transparency and reliability of the qualitative research and the analysis process, four Guba and Lincoln criteria including credibility, transferability, dependability (consistency) and confirmability were used [19]. In order to enhance the credibility of the study, long-term engagement and continuous observation were used, so that the researcher was fully engaged in the study, made proper communication with the participants, and accepted the deeper concepts that emerged in the study process. The method of the combination of interview and literature review was also used to triangulate the data sources and increase the credibility. In this regard, different frameworks of policy analysis were studied and the framework proposed by Matus had the most correspondence with the present concepts. In order to increase the confirmability of the results, the coded data were provided to the participants to confirm the validity and accuracy of the results. In order to improve the transferability of the study results, the conditions of the informed participants of the study and the method of interviewing were clearly defined. There was an attempt to select the sample population based on the purpose of the study without any bias, also, collection and analysis of the data occurred simultaneously, Meanwhile, the researchers tried to be fully aware of the theoretical foundations of the study to increase the transferability. Finally, in order to enhance the dependability of the study results, the process of coding the transcriptions to create concepts and themes, as well as textual and audio information will be available. Also, in order to ensure dependability, two members of the research team individually analyzed the content and discussed agreements and disagreements.

## Results

**Fig. 1:**
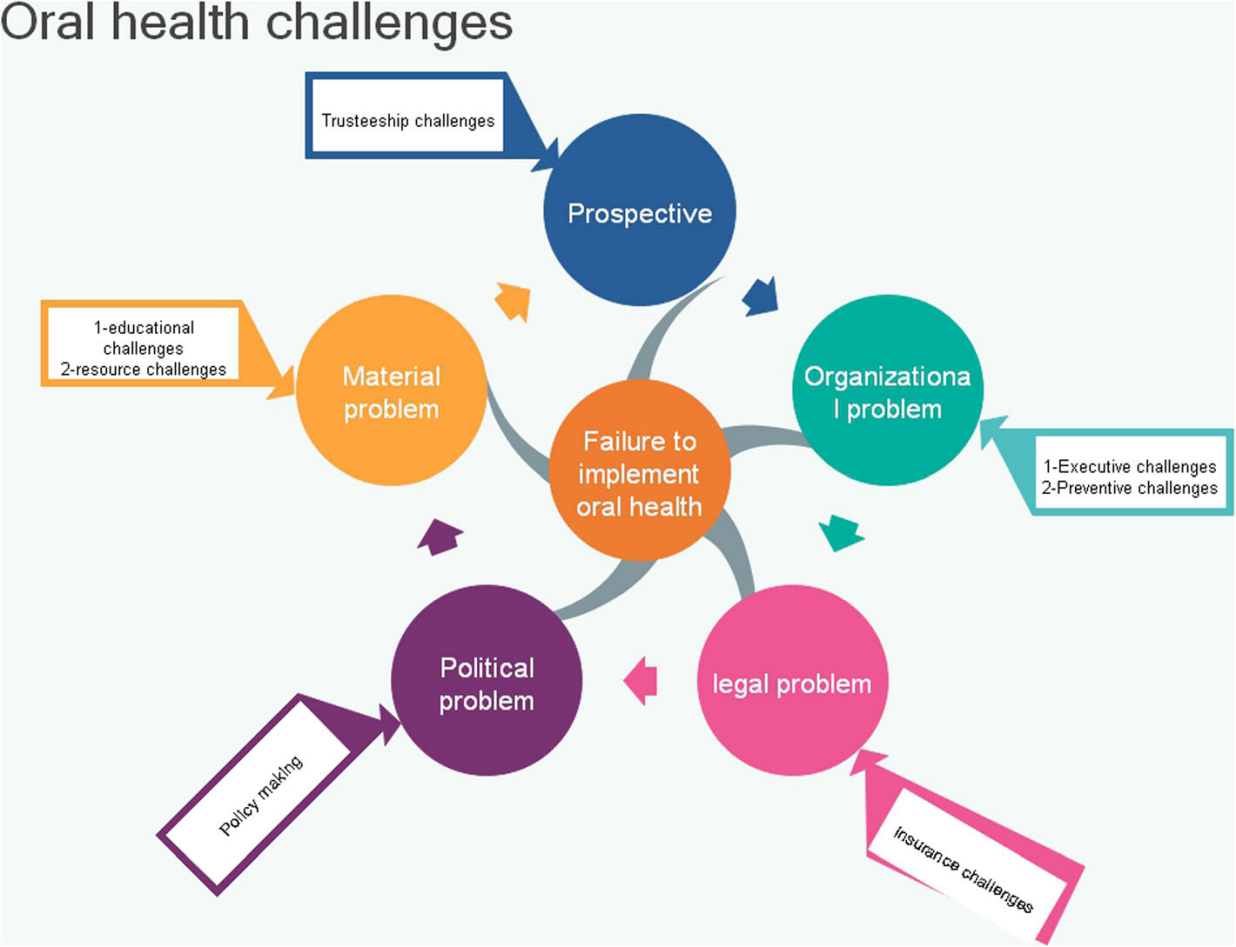
The framework of the Iranian Policy Implementation Challenges

Figure 1 shows the results of the study according to the Matus framework (1996). According to this framework there are 5 main problems in policy implementation that affect the policy products. These five main problems include organizational, material, political and legal problems as well as perspective. The present data analysis extracted from the interviews identified 7 main themes that are situated in the framework (Fig. 1). In this regard, the main themes of executive and preventive challenges to implement oral health policies were categorized as organizational problems, the main themes of educational and resource challenges were situated as material problems, and the main themes of insurance, policy making and trusteeship challenges were considered as the legal, policymaking and perspective categories according to the Matus framework (Fig. 1).

Table 3 indicated that the pre-stated main themes were described with the related 20 sub-themes and some final codes. These three main themes and their subthemes were described as follows:

**Table 3:** Oral Health Policy making Implementation challenges

Main themes	Sub-themes	Final codes
Executive challenges	Health care interventions	Design of therapeutic interventions
		The high cost of treatment centered plans
		The need to design comprehensive and fair plans
		Fair access to services
		Pay attention to prevention in the design of intervention
		Leveling Services
		Considering the cost effectiveness of package design
	Monitoring and evaluation	Lack of cost-effectiveness assessments of oral health plans
		Separation of the evaluation team from the implementation
		Lack of a proper evaluation system
		Lack of a proper monitoring and evaluation protocol
		Problem monitoring due to the complexity of services
	Service delivery	Pay attention to the burden of diseases
		Serious attention to the referral system
		Necessary to design appropriate service structure
		Provide preventive and effective care by intermediate forces
	Oral Health Information System	Inappropriate analysis of oral health state
		Mismatch of statistics and information with existing situation
		Necessity of designing a strong and efficient information system
		Lack of an integrated information system
Prevention challenges	Priority of treatment to prevention	Dentists’ desire for treatment
		More revenue in the field of treatment
		Resource allocation to prevention
		Pay attention to self-care
	Ignore the prevention debate	Not paying attention to prevention
		Design of prevention-based interventions
		Prioritize for prevention
		Lack of prevention attitude in policymakers
		Use inexpensive prevention tools
		Lack of proper prioritization in oral health
		Inadequate understanding of prevention in intervention design and policy making
Educational challenges	Educational curriculum	Treatment-based education curriculum
		The educational curriculum is not community-based
		Need-based curriculum Change
		Attention to prevention in students’ curriculum
	Educational rules	Educational wrong policy making
		Lack of policy-making for oral health education
		Inefficiency of the Human Resources Plan Act
		Strong regulatory for hiring intermediate forces
		Necessity of intervention and implementation of the obligations of trained forces
	Educational infrastructure	Weaknesses in educational need assessment
		Hiring Social Dentistry Graduates
		Declining dental schools
		The cost of undesired effectiveness of increasing dental colleges
		Dental colleges beyond need
		Training of a dental specialist is overly needed
		Convert some colleges to clinics
		Lack of impact of increasing colleges on improving indicators
	Training of allied oral health practitioners	Oral Health worker Education
		Using educational interfaces for schools
		The Cost of training a Dentist
		Effectiveness of allied oral health practitioners
		Low cost of training allied oral health practitioners
		Successful experiences of allied oral health practitioners
Resource challenges	Financial resources	Lack of optimal allocation of funds
		Lack of clear financial resources
	Human resources	Dentist training as needed
		Density of dentists in centers
		HR Needs Assessment
		Improper distribution of dentists
	Physical Resources	Necessary equipment and infrastructure
		Infrastructure and equipment needed in deprived areas
		Lack of infrastructure and facilities at prevention centers
		Infrastructure burnout in deprived areas
Policy making challenges	Lack of policy makers	Lack of policy maker in the field of oral health
		The presence of therapists at the top of policy making
		Non-hire of social dentists
		Weakness in policy making knowledge and health economics among policymakers
		Lack of relevant policymakers
		Neglecting Social Dentistry in Policy Making
		Lack of relevant policymakers
	Evidence-based policy making	The policymaker’s view of dentistry as a luxury service
		The therapeutic approach in policy making
		Designing native health packages
		Lack of evidence-based policymaking
		Lack of awareness of full service package of policy making
		Serious attention to supply and demand in policymaking
	Conflict of interest	Necessity to reduce profession and union look
		Conflict of interest in training intermediate forces
		Conflict of interest in policy making
		Transparency in the public and private sectors
		Protecting corporate interests in the face of wrong measures
Insurance challenges	Unclear laws for identifying target groups	Pay attention to target groups
		High-risk age group coverage
		Lack of coverage for high disease burden age group
		Elderly insurance coverage
	Correction of basic benefit package	Dental services under insurance coverage
		Need to modify basic insurance package
		Expensive services and unwillingness of insurance
		Target groups basic insurance
		Pay attention to the burden of diseases on the insurance package
		Poor insurance coverage
Trusteeship/Stewardship challenge	Unit trusteeship	Multiple trusteeship in the field of oral health
		Necessity of coordination of all three departments of education, health and treatment
		Difficult to enforce policies
		Multiple decision making in the field of oral health
		Single trusteeship with separate experts
		Private sector trusteeship
		Wandering over resources and structure
	Monitoring and coordination	Dividing tasks in the trusteeship
		Appropriate trusteeship and attention to the private sector
		Coordination and monitoring of public and private sectors in service provision
		No oral health plan at the Ministry of Health

### Policymaking challenges

Policymaking challenges included three sub-themes: lack of policymakers, weakness in evidence-based policymaking and conflicts of interest. The participants stated that lack of relevant policymakers in the field of oral health has caused weaknesses and problems in improving oral health. In this regard, a participant stated:

*“After the Islamic Revolution, a number of physicians were specialized and at the same time, became familiar with the management requirements as well as health policymaking and health economics. Unfortunately, such an improvement hadn’t occurred in dentistry. This is our major challenge: no manager and policymaker in the field of oral health” (P*_*1*_*).*^1^

In this regard, another interviewee stated:

*“According to the policies, the aim was to train social dentists who study management and health policymaking and become familiar with public concepts but they come into the field and practice treatment procedures. As a result, when they deal with the duty of community oral health they have no vision of community and health” (P*_*2*_*).*

Another sub-theme of policymaking was the lack of attention to evidence. In this regard, one of the interviewees stated:

*“When we ignore the evidence-based preventive packages to improve oral health today, and turn to restoration packages instead, the oral health won’t improve” (P*_*3*_*).*

In this regard, another interviewee said:

*“In the Ministry of Health plans, Oral Health is ignored. Because the policies of the Ministry of Health do not follow evidence-based policymaking. The policies are passive and because of resource constraints or lack of understanding of complete benefit packages, the Ministry of Health, does not seriously pay attention to dental health or public demand” (P*_*6*_*).*

### Educational challenges

Other major themes in this study were educational challenges in the field of oral health which led to the identification of the sub-themes of “lack of appropriate educational curriculum”; “inefficiency of educational rules”; “educational structure” and “training of intermediate forces”.

Here is the statement of a participant about lack of appropriate educational curriculum:

*“This is a debate in our dental education in another words, dental curriculum is problematic and it is not community-oriented. At the same time, the distribution of the sources is not equitable” (P*_*11*_*).*

In another place, another interviewee said:

*“Educational curriculum is not based on community needs. The curriculum should be changed according to the population conditions such as population aging” (P*_*1*_*).*

One of the sub-themes was the inefficiency of educational rules. In this regard, one of the interviewees stated:

*“For years, the Ministry of Health’s policy was to train some people with lower dental facilities who after graduating, provide dental services in underpriviledged areas the same as rural and urban fringe ones, but due to lack of proper education, this goal has not been achieved” (P*_*12*_*).*

Another sub-theme in educational challenges was training of the oral health care workforce; in this regard one of the participants stated:

*“The initial oral health policies was focused on training of health workers along with dental hygienists, and a lot was spent for this policy; they went to deprived areas; but after some years, the health workers tend to graduate from dentistry schools. Unfortunately, this effective policy did not continue because of the conflict of interest, which means that the dental colleges considered this program [training dental technicians] as a rival and hindered it” (P*_*4*_*).*

### Resource challenges

Resource challenges in oral health policy implementation consist of financial, human and physical resources.

In this regard one of the interviewees stated:

*“At one point, there was a pressure on building a dentistry school in every city. A huge budget that should be spent on preventative dental health had been used for dental colleges, these colleges devour huge budgets every year without any attempt to reach to the national oral health goals” (P*_*1*_*).*

Another interviewee stated:

*“Lack of financial resources allocated to the preventive goals as well as the community oral health specially in underdeveloped regions and rural areas led to failure of the oral health policies …” (P*_*5*_*).*

About the human resource challenges, one of the participants said:

*“Now health care system faced with a shortage of dentists in deprived and underprivileged areas. This shortage is also more significant for oral health technicians in that regions” (P*_*4*_*).*

Another participant emphasized that:

*“In order to promote the oral health, allied oral health practitioners should be trained, such as a dental technician’s, hygienists, etc. These practitioners can be very effective; for example, in many developed countries due to high costs of training dentists, allied oral health practitioners are trained to do the related tasks of preventing, educating and surface restorations that are very effective of course if there is a proper monitoring system” (P*_*7*_*).*

### Executive challenges

The executive challenges were one of the main themes that led to the identification of sub-themes in the field of “service delivery”, “health interventions”, “Oral Health Information System” and “monitoring and evaluation”:

In this regard, one of the interviewees stated:

*“In the provision of dental services, no relationship is found between the private and public sectors, such as public and special problems in this field. Dentistry, as a luxury field of study, has its own costly services and difficulty and easy access is not yet fully established.” (P*_*4*_*).*

Elsewhere, another interviewee said:

*“In service delivery, there should be a leveling service and referral system. If the referral system is implemented, the service delivery will be improved as well, which of course requires allied oral health practitioners to provide basic services; if necessary, referring the patient to the dentist will save money and provide him with timely services” (P*_*3*_*).*

Monitoring and evaluation was another sub-theme stated in this regard:

*“Monitoring and evaluating are very important, especially in national plans, and if the plans are not implemented effectively, due to lack of proper monitoring after implementation, some of the prevention plans that have been implemented so far have failed. For example, dentistry students are sent to schools for education of the children and fluoride therapy. It is really important to have an appropriate monitoring system if we expect good results in practice” (P*_*8*_*).*

In this regard, another interviewee added:

*“Much attention should be paid to the evaluation and monitoring of oral health plans, and the point to be made in this regard is the need for a supervisor and evaluator to be separated, which is unfortunately not the case now” (P*_*9*_*).*

### Insurance challenges

Insurance challenges were another theme of the study that included the sub-themes of “Unclear laws for identifying target groups” and “Correction of basic benefit package”.

In this regard, the participants stated:

*“In many European countries, children are insured since birth, examined every 3 to 6 months until the age of 18, and children and their parents are trained to do preventive activities the same as fluoride therapy or fissure-sealant. These are compulsory interventions just like vaccination even if a person does not refer, the system, follows them. This prevents the burden of treatment. Nowhere else in the world dental insurance coverage is free because expensive services and high costs cannot be insured. The insurance coverage for dental problems may be possible only when we take preventive measures and provide coverage to persons under 12 years of age or surface repair. In Iran, restoration of teeth was implemented for 6 teeth but due to lack of proper supervision they all refer for the restoration of teeth even if it is not required the dentists do it because they receive their per case. This leads to the supply induced demand” (P*_*6*_*).*

### Trusteeship challenges

This theme includes two sub themes of “unit trusteeship” and “Monitoring and coordination”.

In this regard one of the participants said:

*“Coordination is really an important issue. Also it is important to clarify the stewardship of this coordination. It is suggested to manage an office by the Ministry of Health to run all the three sectors and coordinate accordingly. But unfortunately no one evaluated the cost-effectiveness of the funds allocated to preventive, treatment or education sections of oral health” (P*_*2*_*).*

Another interviewee stated:

*“No one is responsible of stewardship. The universities have different practices, in fact, because the principal executives do not hire those who have both knowledge and expertise, so these problems exist” (P4).*

### Prevention challenges

The challenges related to prevention that led to inappropriate implementation of oral health policies were regarded as “priority of treatment to prevention” and “ignore the prevention debate”.

In this regard one of the participants indicated:

*“In the field of health, the attraction of the treatment sector caused an increase in the willingness to treatment and wealth instead of attention to the community’s health. Unfortunately, the highest level of oral health policymaking in the country is done by the specialists, that’s why the prevention is always ignored” (P*_*11*_*).*

Another interviewee mentioned elsewhere:

*“The amount of support for dentistry is not enough, because these services are so expensive and the imposed costs are inevitable for everyone who goes into the treatment cycle. So, the treatment package with a therapeutic approach can` be very effective and it is necessary to have a shift to a preventive approach with a comprehensive monitoring and supervision” (P*_*10*_*).*

## Discussion

The present study was conducted to identify the challenges of oral health policy implementation in Iran. The challenges that were identified included policymaking, executive, education, stewardship/trusteeship, prevention, insurance, and resource allocation.

According to the results, neglecting oral health in policymaking and upstream documentation is one of the challenges in this field. In this regard evidence indicates that attention to oral health for allocating resources and cost-effectiveness of its services is not as much of a concern for health decision makers and policymakers, especially in developing countries [15, 16]. Other evidence shows that ignoring oral health in policymaking of the developing countries` health sectors is considered as a real challenge. This challenge, leads to some degree of failure in these countries to implement a national plan for oral health [20]. Moreover, neglecting oral health policy in these countries may lead to increased out of pocket payments for patients [21]. In spite of the above statements, evidence-based policymaking is one of the key topics highlighted by the World Health Organization. It develops strategies that should be the basis for policymaking in developing countries, such as reducing the burden of oral diseases, improving lifestyles, developing a system of fair service in oral health, and developing a program-based policymaking framework for community health promotion [12].

One of the policymaking challenges in this study was the lack of effective health interventions and dentists’ willingness to treat, as well as the lack of effective policymaking in the field of prevention. The dentists’ willingness to work in the private sector and generate income in this sector is a common theme in most countries [22]. As the evidence shows, in developing countries, applying a preventive approach for all the population is more cost-effective than therapeutic and restorative approaches [23]. In this regard, WHO recommends development of oral health services with a health care approach and to integrate them in a primary health care system*.* In Iran, in spite of preventive policies by the Ministry of Health, as well as some emphasis in national documents, these interventions appear to still have weaknesses, including proper post-implementation evaluation and monitoring. It is essential for the Ministry of Health to adopt effective policies and payment system reform to eliminate this shortcoming.

One of the other challenges identified in this study was the high cost of training general practitioners and specialist dentists and not paying attention to the training of intermediate forces (oral hygienist, oral health care provider, and oral technician). It was found that the policy of increasing the number of dental colleges and training of dental specialists did not lead to improvement of oral health. It is also emphasized that increasing the number of dentistry colleges in developing countries, not only causes high costs for the health care system, but also creates serious concerns about the quality of services and education [24]. Other evidence also suggests that in developing countries the policy of increasing and training intermediate forces can provide better results and help improve the oral health state of the community [23], so that this policy can reduce caries, increase and improve restoration and reduce dental emergencies, especially in children.

According to the results, another challenge was the current educational curriculum, which is not community-based nor based on the epidemiology of oral diseases. Many studies around the world have also emphasized the importance of changing the educational curriculum of dentistry and the need to have goals such as effective prevention, health promotion, high communication skills, and recognition of the social environment [25, 26]. A therapeutic approach in the educational curriculum of dentistry with its over emphasis on therapeutic interventions based on technology instead of attention to preventive education is one of the main challenges and barriers of community based dentistry [27–29]. Revising the educational curriculum of dentistry concerning community needs and socio-economic conditions particularly in developing countries should be carefully considered by their policy makers.

Insurance coverage in oral diseases is a global challenge due to the high cost of services and the luxury nature of many services. Some evidence suggests that insurance coverage emphasizes and prioritizes early prevention and treatment services with a target groups’ priority. It also results in lower cost to the health system and has more health-related consequences [30]. Some developed countries, including Japan, have covered their dental services under their insurance plan and have achieved good results in adopting uniform tariff policies in the private and public sectors [31]. The challenge of access and utilization of oral health services in most developing countries is considered as a serious barrier. For instance, according to a utilization study, the results of implementing oral health programs in European developing countries has indicated that access to oral health services was very low and most of the referrals to dentists were related to extraction of teeth and therapeutic interventions. In contrast, the rate of preventive services` utilization was reported to be less than 10% [32]. However, utilization of oral health services was more based on preventive interventions and insurance coverage can be used as a facilitator in this regard [33]. This topic of insurance coverage for preventive services in developing countries such as Iran is mentioned in the present results too, which could result in better effectiveness.

One of the other challenges identified in this study was resource challenges consisting of human, physical and financial resources. Evidence shows structural problems in developing countries are considered as a serious problem that can have a significant impact on the quality and access to oral health services. So implementing national programs of oral health the same as screening, is mentioned as a high priority concern in these countries [34].

Access to dental services, including fair and equitable distribution of dentists and physical infrastructure especially in the deprived and underprivileged areas, is considered as one of the oral health promotion problems. This phenomenon is named care inversion. Various studies around the world have also addressed this problem of care inversion, and have indicated the need for attention [35, 36]. In this regard, one of the important challenges to reach equitable distribution of services is the number and allocation of the workforce in a delivery system. This is reported as a significant challenge in developing countries. Concerning unfair distribution of services and workforce in deprived and rural areas along with the correlation between socioeconomic factors and access to oral health services, a fundamental change in policymaking is needed in order to achieve equity [14].

One of the problems raised in this study was the weakness of the integrated information system and the accuracy of the data. Previous studies have also emphasized the importance of an integrated system for collecting and analyzing information in this field, which requires serious attention from policymakers [37].

Finally, another challenge discussed in this study was attention to proper stewardship of oral health. It seems that having an integrated stewardship in oral health is an important and influential issue in this field. Previous studies suggest that stewardship should be integrated under the supervision of the Office of Oral Health, so that the tasks of policymaking and enforcing these policies in the public and private sectors are considered [38].

## Conclusion

The study showed that the implementation of oral health policies in Iran has faced some challenges. In this regard, Iranian policymakers and managers need to pay serious attention to the oral health implementation strategies to improve it at the national and regional level. Furthermore, serious review of educational, preventive and therapeutic policy implementation strategies is needed. On the other hand, it seems that national coverage of oral health and the integration of these services in preventive services and serious attention to the private sector can be considered as the most important strategies for achieving improved oral health.

### Limitations

This study has some limitations including lack of triangulation of the viewpoints of the participants with the quantitative indicators, particularly in deprived and rural parts of the country because of the lack of an integrated information system. Also, the results may be useful and applicable only for those in underdeveloped and developing countries with a similar context of health and its determinants such as Iran.

## Data Availability

The datasets used and/or analyzed during the current study are available from the corresponding author on reasonable request.

## Abbreviations

DMFT: Decayed, Missed and Filled Teeth
WHO: World Health Organization
HIV/AIDS: Human immunodeficiency virus infection and acquired immune deficiency syndrome
PHC: Primary Health Care

## Footnotes

1 P_1_ stands for participant number 1. We use the numbers to maintain participant confidentiality
